# Influence of Magnetic Stirring and Eutectic Front Velocity on the Solidified Microstructure of Al-18 wt.% Si Alloy

**DOI:** 10.3390/ma17246029

**Published:** 2024-12-10

**Authors:** Dimah Zakaraia, András Roósz, Arnold Rónaföldi, Zsolt Veres

**Affiliations:** 1 Institute of Physical Metallurgy, Metal Forming & Nanotechnology, University of Miskolc, 3515 Miskolc, Hungary; zakaraia.dimah@uni-miskolc.hu (D.Z.); zsolt.veres@uni-miskolc.hu (Z.V.); 2 HUN-REN TKI, Materials Science Research Group, University of Miskolc, 3515 Miskolc, Hungary; elkronar@uni-miskolc.hu

**Keywords:** hypereutectic Al-Si alloys, RMF, eutectic front velocity, eutectic structure, interlamellar distances

## Abstract

The microstructure of hypereutectic Al-Si alloys is crucial in determining their mechanical properties and overall performance in engineering applications. This paper investigates the effect of a rotating magnetic field (RMF) and eutectic front velocity on the microstructure of hypereutectic Al-18 wt.% Si alloy. The hypereutectic samples were solidified using five different front velocities (0.02, 0.05, 0.09, 0.2, and 0.4 mm/s) with an average temperature gradient (G) of 8 K/mm in a crystallizer equipped with an RMF inductor. The samples were solidified into two sections. The first section solidified without stirring, while the second section solidified with stirring using RMF at an induction (B) of 7.2 mT. The length, angular orientation of eutectic Si lamellas, and interlamellar distances were measured in both the non-stirred and the stirred sections to evaluate the impact of RMF and front velocity on the eutectic structure. The results revealed that the application of RMF and the increase in front velocity during solidification led to the significant refinement of the eutectic structure. These findings highlight the potential of RMF and front velocity manipulation to enhance the microstructure of hypereutectic Al-Si alloys, with practical implications for the development of high-performance materials.

## 1. Introduction

Al-Si alloys are highly valued in engineering for their low weight, robust mechanical performance, excellent thermal conductivity, and strong resistance to corrosion [1]. When these alloys contain more than 12.6 wt.% Si, they exhibit hypereutectic microstructures, characterized by primary Si embedded within an Al-Si eutectic matrix [2]. Hypereutectic Al-Si alloys have gained significant attention in the industrial sector due to their excellent combination of high strength, wear resistance, and thermal stability. However, their microstructure is complex, irregular, and heterogeneous, which affects their mechanical properties. Hard primary Si particles and long eutectic Si lamellas give hypereutectic Al-Si alloys high strength and wear resistance but also result in low ductility and poor machinability, severely limiting their application in promising sectors [3]. In addition, the long eutectic Si lamellas and large primary Si blocks act as a stress concentration point for cracks, causing these alloys to suffer from brittleness and making them prone to fracture [4]. The microstructure of these alloys strongly influences their mechanical properties; thus, controlling their microstructure is essential for achieving the desired mechanical performance. A widely used approach to enhance the microstructure and properties of these alloys is to alter the solidification conditions, such as by controlling the eutectic front velocity or applying melt conditioning methods like inducing the melt using a rotating magnetic field (RMF).

The rotating magnetic field (RMF) is a powerful tool used to control melt flow during solidification processes in materials science. It induces two distinct types of melt flow during solidification: azimuthal and axial flows [5,6]. Azimuthal flow is perpendicular to the axis of the solidification direction, while axial flow runs parallel to it, causing the melt to flow from the hotter to the colder regions of the crucible. These flows can be beneficial for removing impurities, promoting the growth of defect-free crystals, and improving the quality of the resulting material. However, they can also lead to undesirable effects such as macro-segregation and non-uniform growth [7].

As a result of the combination of the azimuthal and axial flows, the motion becomes spiral, which substantially influences the distribution, concentration, and growth morphologies of alloying elements [8]. Both azimuthal and axial flows can be controlled and optimized by adjusting the parameters of the RMF. By harnessing RMF to control melt flow, researchers can improve the quality and performance of various materials, ranging from metals and alloys to semiconductors and biomaterials.

Several studies [8,9,10] have investigated the effect of RMF on the microstructure of metallic alloys, with most finding that RMF-induced melt flow leads to a more refined microstructure and improved mechanical properties. One study by Al-Omari et al. [8] experimentally examined the impact of RMF on the microstructure of an Al-12.6 wt.%-Si alloy. The samples were unidirectionally solidified under different magnetic induction levels at a front velocity of 0.1 mm/s and a temperature gradient of 6 K/mm. The study showed that RMF significantly affected the shape and distribution of the eutectic Si in the solidified structure. Specifically, the authors observed a finer eutectic structure in the samples subjected to forced melt flow than those not.

Jie et al. [9] found that applying RMF during solidification significantly influences the structure of hypereutectic Al-Si alloy. The primary Si tends to separate and distribute uniformly along the radial direction of the sample, resulting in a more refined primary Si in the final microstructure. The researchers found that this phenomenon is attributed to the Lorentz force generated by the interaction between the rotating magnetic field and the eddy currents, inducing convective motion and promoting the separation of the primary Si from the melt.

Another study by Yan et al. [10] demonstrated that RMF generates a stirring effect, disrupting the dendritic network in the CuNi10Fe1Mn alloy, which enhances nucleation and increases the formation of fine, equiaxed grains. The researchers also reported that RMF improved the mechanical properties of the CuNi10Fe1Mn alloy.

In addition to applying RMF, controlling the eutectic front velocity during solidification is another method for refining the microstructure of metallic alloys. Numerous studies have examined the impact of front velocity on the eutectic microstructure. Jackson and Hunt (J-H model) established a generic quantitative equation based on a diffusion problem at the eutectic front, which is λ^2^v = const, where λ is the eutectic interlamellar distance and v is the front velocity [11]. They concluded that the eutectic interlamellar distance is an important microstructural parameter for eutectic alloys and is affected by the front velocity. Zuo and his colleagues [12] investigated how front velocity and external magnetic fields affect the structure and mechanical characteristics of Ag-Cu eutectic alloys and found that increasing front velocity led to a more refined microstructure and improved mechanical properties.

In a Bridgman furnace, Koçak et al. [13] unidirectionally solidified a Bi-Pb alloy at the eutectic composition in the upward direction at five different front velocities, maintaining constant temperature gradients. Their research showed that as front velocity increased, the interlamellar distance decreased, and microhardness increased for a given G.

Overall, these studies indicate that forced melt flow by RMF and controlling the solidification front velocity significantly impact the microstructure of metallic alloys.

Recent advancements have leveraged synchrotron X-ray radiography and tomography to study the operando growth dynamics of primary Al_3_Ni intermetallic phases during the solidification of Al-15 wt.% Ni alloys under magnetic pulses up to 1.5 T. These real-time observations, complemented by a multiphysics numerical model, quantified the differential forces and stresses influencing crystal growth, branching, and fragmentation. This work provides a robust theoretical framework for understanding intermetallic phase dynamics and fragmentation mechanisms under pulsed magnetic fields, addressing longstanding hypotheses in the field [14].

However, further investigation is needed into the RMF and front velocity conditions for hypereutectic Al-Si alloys and their effect on the microstructure of these alloys. Our investigations fit in the range of the studies so far. This paper aims to contribute to this research by further investigating the impact of forced melt flow using RMF and front velocity on the eutectic lamellas of hypereutectic Al-18 wt.% Si alloy. The eutectic Si lengths, their orientation angles relative to the solidification direction, and the distance between Si lamellas were measured as a function of front velocity with and without stirring by RMF. A hypereutectic Al-18 wt.% Si alloy was chosen for investigation in this study because the primary Si can grow freely without touching each other. This study is part of the international MICAST project, which utilizes this alloy.

## 2. Materials and Methods

### 2.1. Materials and Experiments

Unidirectional solidification was conducted on Al-18 wt.% Si hypereutectic samples. The diameter of the samples was 7.1 mm, and their length was 110 mm.

The solidification was performed using the vertical upward Bridgman technique, a technique for solidifying materials in a controlled manner [15]. This method involves heating a sample above its melting point and then slowly lowering it into a cooler zone. As the sample moves downward, it solidifies from the bottom up. The solidification was conducted using a Crystallizer with a High Rotating Magnetic Field (CHRMF) device, which controls the temperature and gradient of the sample during solidification (The equipment and all of its parts are developed by our research group and the developer is Arnold Rónaföldi, Miskolc, Hungary, EU) [16]. This device was used to create a nearly uniform temperature gradient across the sample, which is important for studying the solidification behavior of the material. Using the vertical upward Bridgman method and the CHRMF device allowed for precise control over the solidification process and helped to ensure accurate and reliable results.

**Figure 1:**
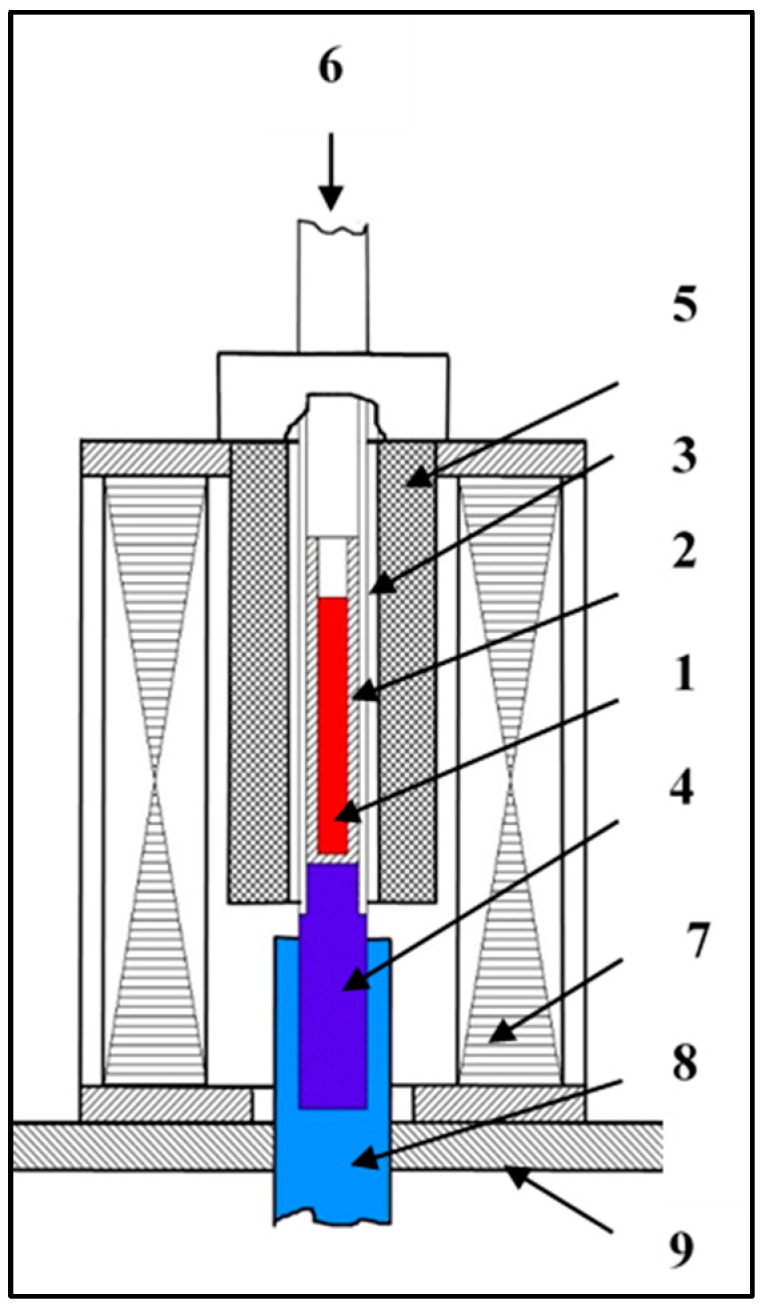
Diagram of the solidification setup: 1: Sample; 2: alumina capsule; 3: quartz tube; 4: copper cooling core; 5: furnace with four separate heating zones; 6: step motor; 7: RMF inductor; 8: water cooling unit; 9: base [7].

The RMF device used in this study consisted of several key parts that work together to facilitate the solidification process (The RMF and all of its parts are developed by our research group and the developer is Arnold Rónaföldi, Miskolc, Hungary, EU) [7,16,17]: an alumina capsule in which the sample solidifies, thirteen K-type thermocouples of varying lengths positioned around the sample, a quartz tube to encase the thermocouples, a copper cooling core, a vertical Bridgman furnace with four separate heating zones, a control unit to manage the transfer of samples from the melting furnace to the cooling unit, a step motor to regulate the sample movement velocity, a MagnetoHydroDynamic stirring (MHD) inductor to generate a RMF during solidification (The MHD inductor is developed by our research group and the developer is Arnold Rónaföldi, Miskolc, Hungary, EU), a water cooling unit, and a basement (Figure 1).

For the experiment, five samples of hypereutectic Al-18 wt.% Si alloy were solidified at five different front velocities: 0.02 mm/s, 0.05 mm/s, 0.09 mm/s, 0.20 mm/s, and 0.40 mm/s, to examine how front velocity influences the microstructure of the alloys. The average temperature gradient (G) was 8 K/mm. Each sample was solidified unidirectionally into two sections: the first half (54 mm long) without magnetic stirring, and the second half with magnetic stirring under a rotating magnetic field (RMF) with an induction of B = 7.2 mT. RMF was applied during solidification to explore the impact of forced melt flow on the microstructure. The front velocity (v) and temperature gradient (G) were determined from the measured cooling curves.

### 2.2. Investigation of the Eutectic Structure of the Solidified Samples

The macrostructure and microstructure of the samples were analyzed using optical microscopy (OM). The samples were ground, polished, and etched in a 2-vol% aqueous HF solution to reveal the microstructure. To characterize the eutectic structure in the Al-18 wt.% Si samples, measurements were taken of the average eutectic Si lamella length, the angle between the axis of the eutectic Si lamella and the direction of solidification, and the average eutectic interlamellar distance (λ).

The image analysis software ImageJ (Image J 1.52a, National Institutes of Health, LOCI, USA) was used to quantify the length and distribution of the eutectic Si lamellas in the microstructure. The length of the Si lamellas was measured using the maximum Feret diameter of each eutectic Si lamella. The length measurements were analyzed for over 200,000 lamellas per sample by classifying the eutectic Si lamella’s length into different ranges, ranging from (0–10) to (90–100) µm. For example, lamellas whose lengths fell between 0 and 10 µm were categorized into one group, those between 10 and 20 µm into another group, and so on, until reaching lamellas whose lengths ranged from 90 to 100 µm. This procedure was applied to both the stirred and non-stirred sections of the five samples.

**Figure 2:**
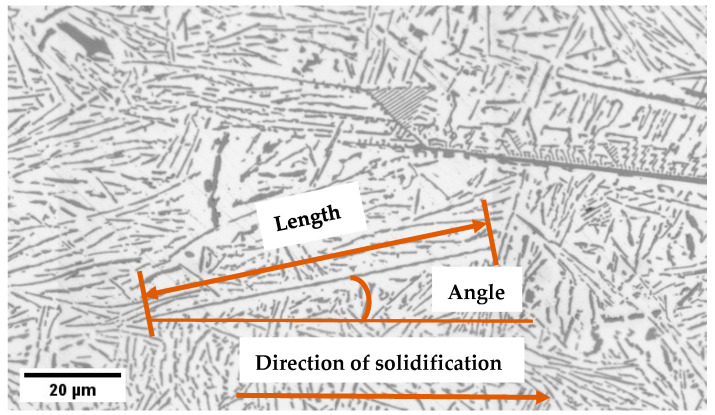
Characterization of the eutectic part in the Al-Si hypereutectic alloy with different characteristics.

The angle between the axis of the eutectic Si lamellas and the direction of solidification movement was measured as a Feret angle [18]. Figure 2 shows the studied alloy’s characterization method of eutectic lamellas. To study the effect of front velocity and RMF on the eutectic Si lamellas’ angles, the measurements were analyzed using a method developed by our research group [9,10]. (i) The lengths of the Si lamellas were categorized into four groups: (0–2), (2–5), (5–50), and (>50) µm. (ii) The orientation of each Si lamella in each length category was measured using the ImageJ software. (iii) The percentage of Si lamellas with the same angle was then calculated for both the stirred and non-stirred sections (iv) Finally, an angle distribution curve was plotted for each length category for both the stirred and non-stirred sections of the samples.

The eutectic interlamellar distance (λ) was measured by the ‘specific perimeter method (SPM)’, developed by our research team. They created an equation that determined the distance between lamellas and proved its validity [18]. The interlamellar distance (λ) can be determined using Equation (1).

(1) $$\lambda =2\times \frac{A_{p\;}\times (1-A_{f\;})}{N\times P_{0}}$$

where

$P_{0}$: average perimeter of the eutectic Si lamella in the examined optical microscope images (µm);
N: number of eutectic lamellas;
$A_{p}$: (area of the picture − area of the primary Si) (μm^2^);
$A_{f}$: area fraction of the eutectic lamellas in the hypereutectic structure.

When measuring the length of lamellas, there is a possibility of a failure margin of ±1 pixel on both ends. Given that 1 micron equals 5.34 pixels by the used magnification, this potential error could result in a length discrepancy of approximately ±0.37 microns. Therefore, in the reported measurements, the detected length may vary by this margin due to pixel-level inaccuracies.

## 3. Results and Discussion

### 3.1. Qualitative Analysis of the Solidified Sample

**Figure 3:**
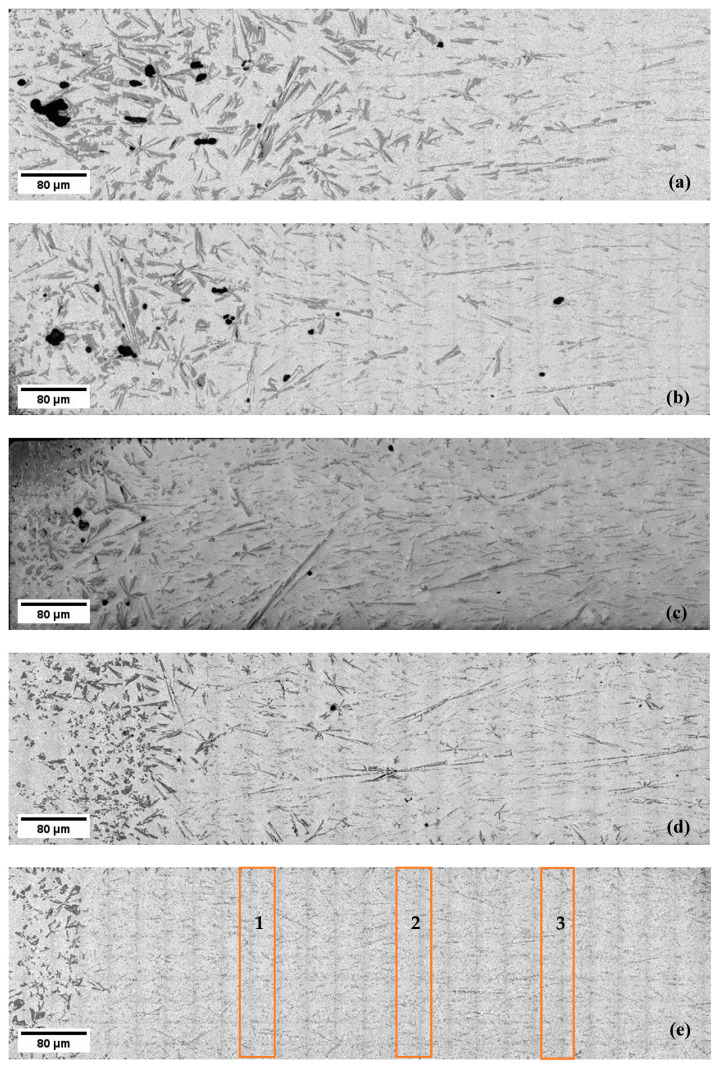
The macrostructure of the non-stirred sections of the hypereutectic samples at different front velocities: (**a**) 0.02, (**b**) 0.05, (**c**) 0.09, (**d**) 0.2, and (**e**) 0.4 mm/s. The measurements of the lengths, angles, and interlamellar distances of the eutectic lamellas were performed in three regions: (1), (2), and (3).

**Figure 4:**
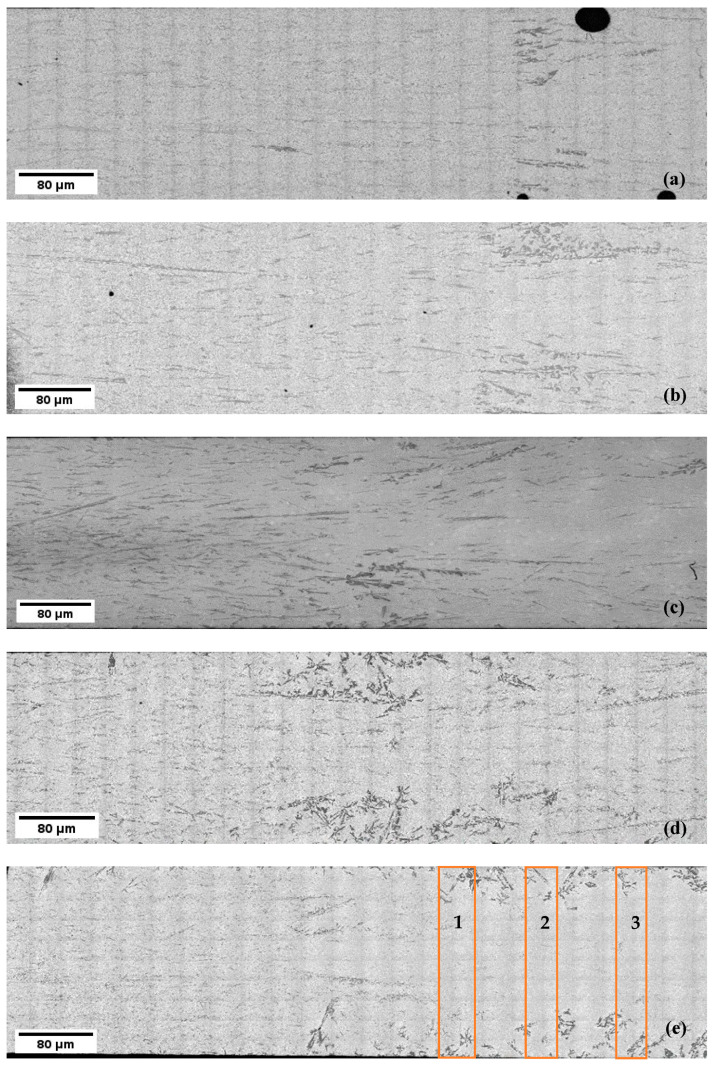
The macrostructure of the transition zone of the hypereutectic samples at different front velocities: (**a**) 0.02, (**b**) 0.05, (**c**) 0.09, (**d**) 0.2, and (**e**) 0.4 mm/s. The measurements of the lengths, angles, and interlamellar distances of the eutectic lamellas were performed in three regions: (1), (2), and (3).

The solidified samples exhibit a macrostructure that reveals a non-homogeneous distribution of primary Si particles in the eutectic matrix, indicating the presence of macro-segregation (Figure 3 and Figure 4). In both the non-stirred and stirred sections of all the samples, differences in the length and shape of the primary Si particles were observed. In the sample solidified at a velocity of 0.02 mm/s, the first half of the non-stirred section showed a higher percentage of primary Si, with larger primary Si particles at the beginning that decreased in size towards the end (Figure 3a). In contrast, in the stirred section, due to magnetic stirring, most of the primary Si particles moved from the central axis to the edges. This resulted in a macrostructure where most of the primary Si solidified at the edges, while the majority of the eutectic matrix solidified in the central region (Figure 4a). These phenomena were also observed in the samples with higher front velocities.

**Figure 5:**
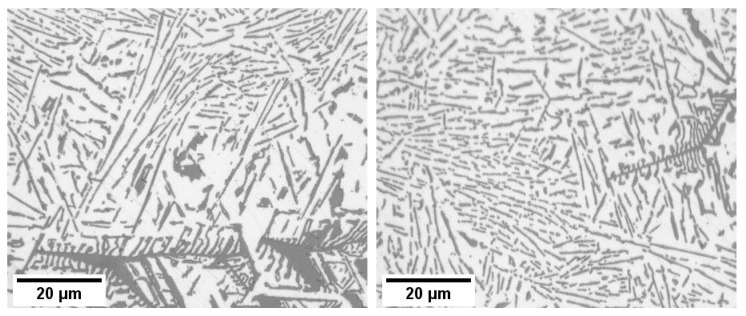
Microstructure comparison of the stirred and non-stirred sections of the sample solidified at 0.1 mm/s. The (**left side**), representing the non-stirred section, shows a coarse eutectic structure, while the (**right side**), from the stirred section, exhibits a finer eutectic structure.

**Figure 6:**
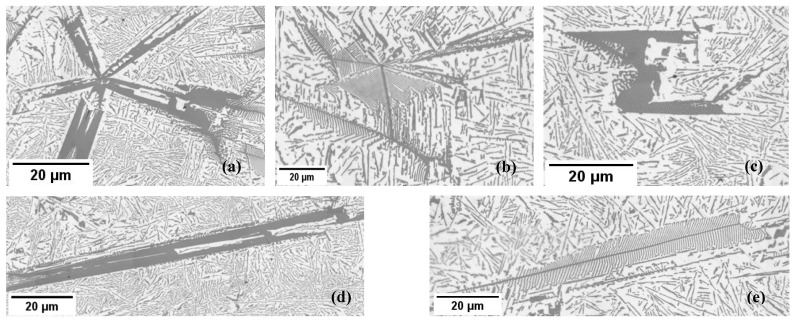
The morphologies of the primary Si in the studied hypereutectic alloy: (**a**) star-like structure, (**b**) dendritic structure, (**c**) polyhedral structure, (**d**) coarse plate-like structure, and (**e**) feather-like structure. The presented microstructures were taken from both the stirred and not stirred sections of the samples.

Further qualitative analysis revealed that the size of eutectic Si lamellas decreases when RMF is applied during solidification (Figure 5). Both the non-stirred and stirred sections exhibited complex and irregular primary and eutectic Si particles, with diverse growth morphologies of the primary Si, including star-like, dendritic, polyhedral, coarse plate-like, and feather-like structures (Figure 6). The eutectic Si lamellas exhibited a plate-like shape. Measurements of the lengths, angles, and interlamellar distances of the eutectic lamellas were performed in three different regions in the non-stirred section and three different regions in the stirred section (Figure 3e and Figure 4e). The rectangles visible in the images resulted from the uneven illumination of the sample during the creation of the mosaic pictures and do not represent elements of the microstructure.

### 3.2. Quantitative Analysis of the Solidified Sample

#### 3.2.1. Length of Eutectic Lamellas

**Figure 7:**
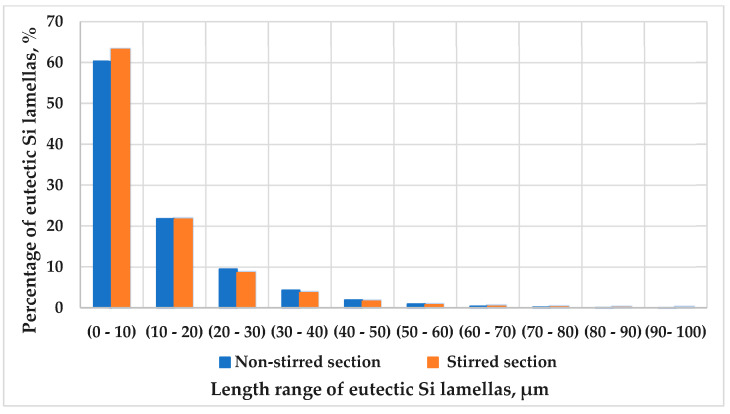
The percentage of the eutectic Si lamellas as a function of their length range in the stirred and non-stirred sections of the sample that solidify at v = 0.02 mm/s.

As outlined in the quantitative analysis, the lengths of the Si lamellas were determined by categorizing their lengths into ranges from (0–10) to (90–100) µm. Figure 7 shows the percentages of the eutectic lamellas in both the stirred and non-stirred sections of the sample solidified at a front velocity of 0.02 mm/s. Although we display only this sample, the trend is consistent across all the samples. The results indicate that at a front velocity of 0.02 mm/s, the percentage of the eutectic Si lamellas decreases as the lamella length increases in both the stirred and non-stirred sections. The same trend was observed at the other applied front velocities.

**Figure 8:**
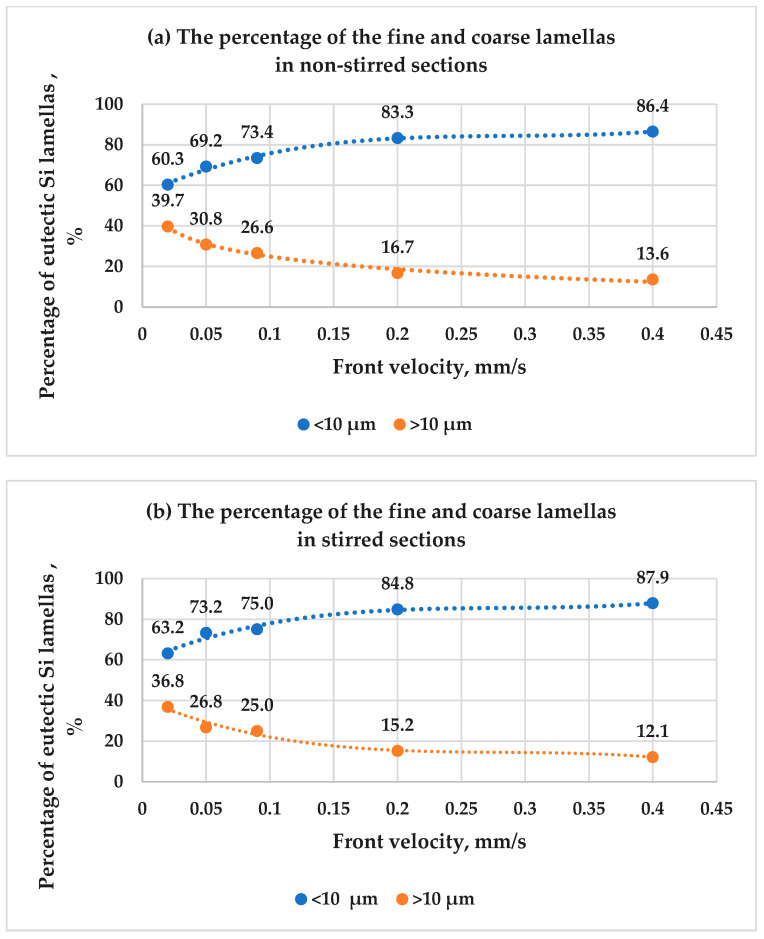
The percentages of the eutectic Si lamellas as a function of front velocity in both the (**a**) non-stirred and (**b**) stirred sections of the five samples.

The eutectic Si lamellas were categorized into two groups: fine lamellas, with lengths less than 10 µm, and coarse lamellas, with lengths greater than 10 µm. This classification was applied to both the stirred and non-stirred sections of all the samples. Figure 8 illustrates the percentage distribution of the lamellas as a function of front velocity in both sections. The microstructure of the hypereutectic Al-Si alloys was strongly affected by increasing front velocity and the application of RMF. The results show that for all the front velocities, the percentage of the fine eutectic Si lamellas was higher than that of the coarse lamellas in both the stirred and non-stirred sections. Furthermore, increasing the front velocity from 0.02 mm/s to 0.4 mm/s led to a higher percentage of fine lamellas and a lower percentage of coarse lamellas in both sections. This is attributed to the shorter solidification time at higher front velocities, which results in a finer microstructure.

It has also been found that stirring the melt using RMF increased the percentage of fine lamellas and decreased the percentage of coarse lamellas compared to the non-stirred sections. This confirms that RMF leads to the refinement of the length of eutectic lamellas in Al-18 wt.% Si samples. These image analysis measurements of the eutectic Si lamellas align well with the microscopic observation (Figure 3, Figure 4 and Figure 5).

The combination of increasing front velocities and the use of RMF during unidirectional solidification has been shown to synergistically affect the refinement of the eutectic structure in hypereutectic alloys. Increasing the front velocities and using RMF promote the formation of a fine eutectic structure due to the limited diffusion of the solute atoms, resulting in a higher density of nucleation sites for the eutectic structure, resulting in a highly refined microstructure.

#### 3.2.2. The Orientation of the Eutectic Lamellas

**Figure 9:**
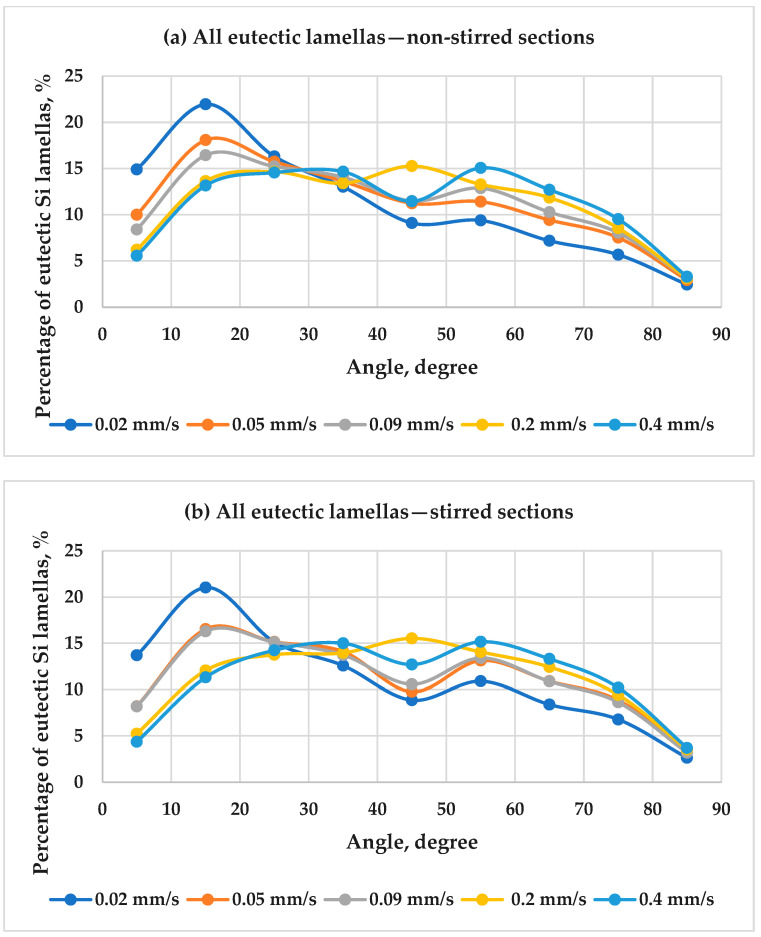
The angle distribution of all the eutectic Si lamellas as a function of front velocity in the hypereutectic alloy for the (**a**) non-stirred sections and (**b**) stirred sections.

The angles of the eutectic lamellas with the solidification direction were determined as the Feret angle. Figure 9 shows the angle distribution of all the eutectic Si lamellas as a function of front velocity in the hypereutectic alloy for both the stirred and non-stirred sections. The analysis highlights the impact of front velocity on the angle distribution. As the front velocity increases, there is a noticeable decrease in the percentage of eutectic Si lamellas with angles less than 25°. Conversely, there is an increase in the percentage of lamellas with angles greater than 25°. This suggests that higher velocities cause a shift toward greater angles, indicating a more varied orientation distribution.

**Figure 10:**
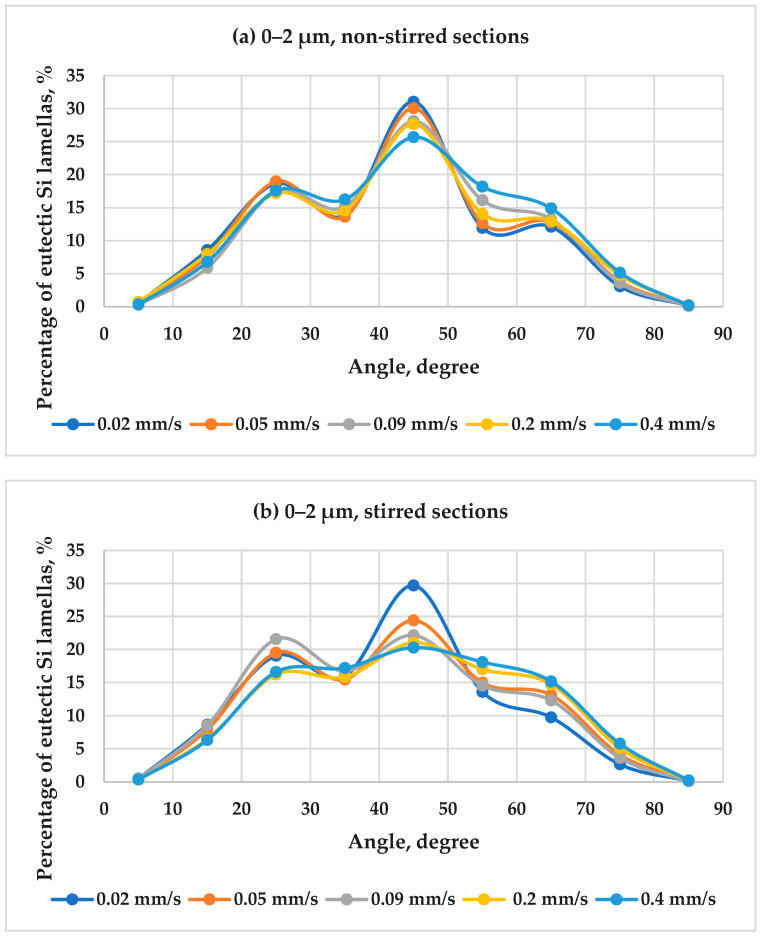
The angle distribution of the eutectic Si lamellas with length 0–2 µm as a function of front velocity in both the stirred and non-stirred sections of the samples.

The front velocity plays a crucial role in the solidification process. It determines the rate at which the eutectic lamellas grow and influences their angle. The orientation of the eutectic lamellas was determined by categorizing Si lamellas lengths into four groups: 0–2 µm, 2–5 µm, 5–50 µm, and >50 µm, followed by computing the angle distribution as mentioned in the quantitative analysis. Figure 10 shows the angle distribution of the eutectic Si lamellas with lengths 0–2 µm as a function of front velocity in both the stirred and non-stirred sections of the samples. The results show that the highest percentage of eutectic lamellas measuring 0–2 µm in length tends to align at a 45° angle to the direction of solidification across all the front velocity values, in both the stirred and non-stirred sections. As the front velocity increases, the percentage of the eutectic lamellas whose length is between (0–2 µm) decreases at its preferred angle in both the stirred and non-stirred sections. The percentage of the lamellas in this range with an angle of about 45° in the non-stirred sections decreases from 31.0% to 25.7% while the front velocity increases from 0.02 to 0.4 µm. The same trend was observed in the stirred sections.

On the other hand, RMF has been shown to affect the orientation of the eutectic lamellas significantly. RMF can induce a rotating flow in the melt, which can modify the solidification front and, consequently, affect the orientation of the eutectic lamellas. Comparing between non-stirred and stirred sections, it has been observed that when RMF is applied, the eutectic lamellas’ maximum percentage (0–2 µm) at its preferred angle (45°) decreases. The rest of the lamellas in this length category are oriented at various angles. The trend was observed for all the values of front velocity.

Inducing melt flow by RMF with increasing the front velocity during solidification decreases the lamellas’ maximum percentage (0–2 µm) at its preferred angle (45°). It increases the angles’ diversity more compared to the non-stirred sections. This means that applying RMF with increasing the front velocity during solidification improves the angle distribution of the eutectic Si lamellas compared to the non-stirred sections.

**Figure 11:**
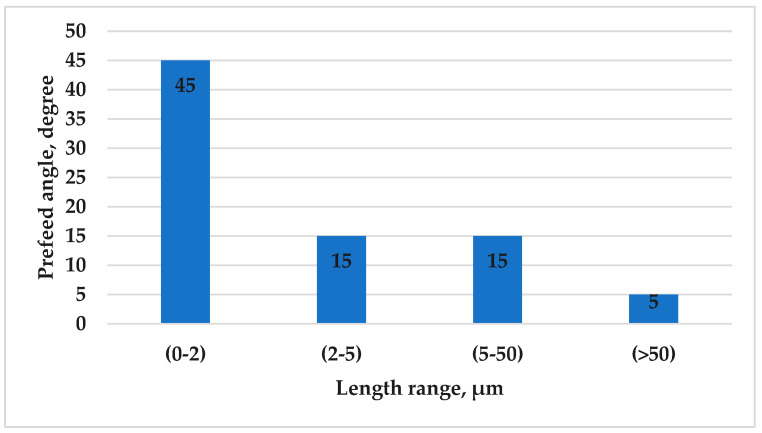
The preferred angle of the eutectic Si lamellas in the hypereutectic alloy for different length ranges.

As previously noted, the preferred angle orientation for eutectic lamellas (0–2 µm) is 45°. Through the same measurement approach, we evaluated the preferred angles across the other different size categories. Notably, we observed that for eutectic lamellas (2–5 µm), (5–50 µm), and for lamellas with lengths greater than 50 µm, the preferred angle orientation is 15, 15, and 5, respectively, at all the applied values of the front velocity in both the stirred and non-stirred sections. The same happened with these categories (2–5 µm), (5–50 µm), and >50 µm; also, increasing the front velocity from 0.02 mm/s to 0.4 mm/s and applying RMF during solidification did not alter the preferred angle of the lamellas, but it did reduce the maximum percentage of lamellas at these preferred angles. This reduction is accompanied by an increase in the percentage of lamellas at other angles. Based on these findings, it was observed that eutectic lamellas that are more parallel to the direction of solidification have a higher likelihood of growing to a greater length (Figure 11).

**Figure 12:**
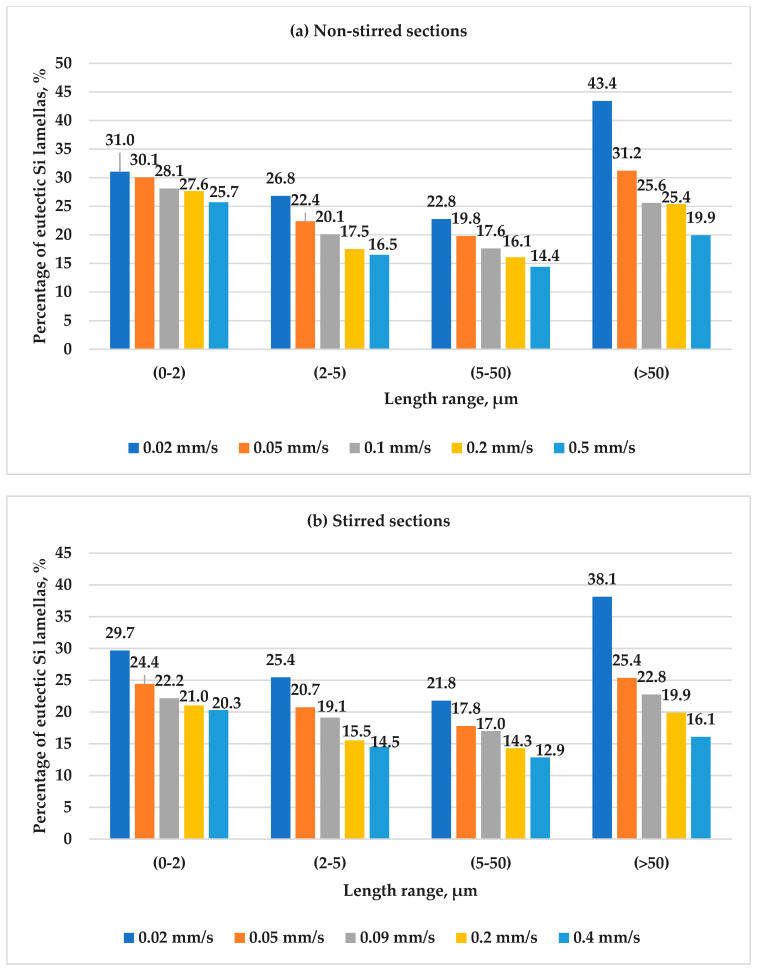
The highest percentages of lamellas oriented at the preferred angle for different length ranges, 0–2 µm, 2–5 µm, 5–50 µm, and >50 µm at different front velocities: 0.02, 0.05, 0.09, 0.2, and 0.4 mm/s in the (**a**) non-stirred sections and (**b**) stirred sections of the samples.

In conclusion, Figure 12 illustrates the variation in maximum percentages across size categories in the stirred and non-stirred sections. Notably, increasing the front velocity and applying RMF during solidification led to a decrease in these maximum percentages. This reduction at the preferred angle corresponds to proportional increases at other angles. Consequently, it can be inferred that increasing the front velocity and applying RMF increases the dispersion of the eutectic lamellas, a trend observed consistently across all the size ranges.

#### 3.2.3. Eutectic Interlamellar Distances (λ)

**Figure 13:**
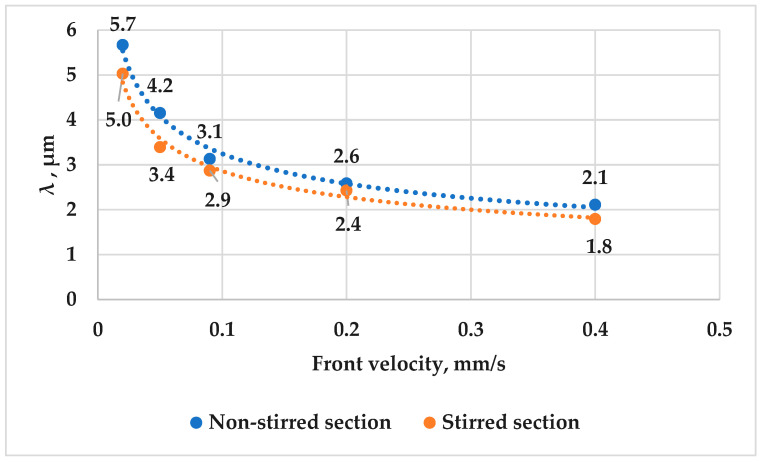
Distance between the eutectic Si lamellas (λ) as a function of front velocity in the stirred and non-stirred sections of the samples.

The results of this study provide important insights into the effects of front velocity and RMF on the eutectic interlamellar distances in hypereutectic Al-18 wt.% Si alloy during solidification. As noted in the quantitative analysis, the interlamellar distances were measured using the specific perimeter method. Figure 13 shows the measured eutectic interlamellar distances as a function of front velocity in both the stirred and non-stirred sections. The study found that increasing front velocity decreased the distance between the eutectic lamellas in both sections. Specifically, in the non-stirred section, the interlamellar distance decreased from 5.7 µm to 2.1 µm as the front velocity increased from 0.02 mm/s to 0.4 mm/s. A similar trend was observed in the stirred section.

Additionally, applying RMF during solidification further reduced the eutectic interlamellar distance compared to the non-stirred sections. Notably, the average distance between the eutectic lamellas in the non-stirred section (5.7 µm) was higher than that in the stirred section (5.0 µm) at a front velocity of 0.02 mm/s, and this trend persisted at higher front velocities. These findings confirm the refinement effect of RMF on the length of eutectic Si lamellas in Al-18 wt.% Si hypereutectic alloy samples. The combination of increasing the front velocities and using RMF during unidirectional solidification has been shown to have a synergistic effect on decreasing the eutectic interlamellar distance and refinement of the eutectic structure in hypereutectic alloys.

These results have significant implications for materials science and engineering, as the distance between eutectic lamellas is a key microstructural parameter that can impact the mechanical properties of alloys. A smaller lamellar distance is associated with improved mechanical properties, such as increased strength and microhardness. These results agree with the results obtained by researcher Koçak and his colleagues [13], reinforcing the understanding of the relationship between front velocity and interlamellar distances in metallic alloys.

The data in Figure 13 provide the following equations:

- For non-stirred sections:

$$\lambda =14.11v^{-0.31}\;\;\;(for\;constant\;G)$$

- For stirred sections:

$$\lambda =11.98v^{-0.31}\;\;\;(for\;constant\;G)$$

The λ value exhibits an exponential dependence on front velocity, with an exponent of −0.31. This exponent is higher than the predicted value of −0.50 according to the J-H eutectic theory [11]. It is also smaller than the values of −0.49, and −0.51 obtained by Koçak [13] for Bi-Pb eutectic alloy, and Kaya and his colleagues [19] for Al-Ni eutectic alloy. These differences could be attributed to the variations in solidification conditions and compositions. Furthermore, these differences may arise from structural discrepancies, where the hypereutectic Al-18 wt.% Si alloys exhibit an irregular eutectic structure compared to other alloys.

For further analysis, it is valuable to consider how different casting techniques impact the solidification process and enhance the temperature gradient, which plays a crucial role in controlling microstructural properties. Different casting techniques have been employed to improve temperature gradients during solidification and refine the microstructure. Reports have shown that adding elements such as phosphorus [20], strontium [21], scandium [22], and boron [23] during solidification can achieve a fine-grained structure. Additionally, the use of a magnetic field [24], ultrasonic vibrations [25], and electromagnetic stirring [26] also have been recognized for their ability to influence the flow of molten metal, enhance the temperature gradient, and refine the microstructure of hypereutectic Al-Si alloys.

The results of this paper agree with the results of Al-Omari [8]. Their experimental research shows that the microstructure of Al-12.6 wt.% Si eutectic alloys changes noticeably when RMF is applied during solidification. Specifically, RMF improves the eutectic structure by decreasing the length of the eutectic Si lamellas, decreasing interlamellar distances, and increasing the variety of orientations of the Si lamellas angle. Al-Omari noted in their study that refinement occurs due to fragmentation. The fragmentation of large Si lamellas leads to the formation of new, finer Si eutectic nuclei that grow in the same direction as the original lamellas, but with a reduced number of lamellas oriented in the preferred growth angle, especially when RMF is applied, resulting in size refinement and a decrease in the preferred angle-orientation percentage.

Jie and his colleagues [27] mentioned in their study that the mechanism behind size refinement is attributed to the increase in the solidification rate, which occurs due to the movement of the cooled liquid from the mold’s perimeter toward the hotter regions.

The observed effects of front velocity and RMF on alloy microstructure in our study can be explained by their influence on the growth process during solidification, where increasing front velocities or applying RMF during solidification leads to accelerated arms growth and competition with each other for space, enhanced natural convection, and limited solute diffusion in front of the liquid–solid interface, resulting in a refined microstructure with closer eutectic lamellas and increased lamella diversity.

## 4. Conclusions

The effect of front velocity and RMF on the microstructure of hypereutectic Al-18 wt.% Si alloy was studied by solidifying five samples with different eutectic front velocities and at a temperature gradient of G = 8 K/mm. Each sample was solidified unidirectionally into two sections: one section without stirring and the other with stirring by RMF at a magnetic induction of B = 7.2 mT. The study involved measuring the length, angular orientation, and distance between the eutectic Si lamellas in both the stirred and non-stirred sections. The results demonstrated that the forced melt flow induced by RMF, and front velocity significantly influence the microstructure of Al-18 wt.% Si hypereutectic alloys.

- Increasing the front velocity during solidification refines the length of eutectic Si lamellas by raising the percentage of fine eutectic lamellas (less than 10 µm) and reducing the percentage of coarse eutectic lamellas (greater than 10 µm).
- Based on the angle measurements, the smallest eutectic Si lamellas (0–2 µm in length) are most oriented at a 45° angle. In contrast, larger eutectic lamellas tend to grow more parallel to the solidification direction. Increasing the front velocity during solidification decreases the maximum percentage of eutectic lamellas at their preferred angles and increases the percentage of lamellas at other angles, resulting in greater scattering of the eutectic Si lamellas.
- Increasing the front velocity during solidification reduces the distance between eutectic lamellas.
- The final alloy’s microstructural evolution is also significantly altered due to RMF, which creates a spiral flow in the melt. The spiral flow modifies heat and mass transfer conditions within the melt and limits the solute diffusion in front of the liquid–solid interface. It, therefore, leads to refining the eutectic Si lamellas, increasing their diversity, and decreasing the distance between them.
- The combination of increasing front velocities and using RMF during unidirectional solidification has been shown to have a synergistic effect on decreasing the eutectic interlamellar distance, increasing the scattering of the eutectic Si lamellas, and the refinement of the eutectic structure in hypereutectic Al-18 wt.% Si alloy samples.
- The findings of this study provide important insights into optimizing microstructural parameters for developing alloys. By controlling front velocity and applying RMF, it is possible to tailor the microstructure of alloys used in various industries, from aerospace to automotive engineering, to achieve improved mechanical properties.

## Data Availability

The original contributions presented in the study are included in the article, further inquiries can be directed to the corresponding author.
